# Synthetic Diphenylacetylene-Based Retinoids Induce DNA Damage in Chinese Hamster Ovary Cells without Altering Viability

**DOI:** 10.3390/molecules27030977

**Published:** 2022-02-01

**Authors:** Lina Hudhud, David R. Chisholm, Andrew Whiting, Anita Steib, Krisztina Pohóczky, Angéla Kecskés, Éva Szőke, Zsuzsanna Helyes

**Affiliations:** 1 Department of Pharmacology and Pharmacotherapy, Medical School & Szentágothai Research Centre, University of Pécs, H-7624 Pécs, Hungary; l.hudhud81191@gmail.com (L.H.); steib.anita88@gmail.com (A.S.); pohoczkykriszti@gmail.com (K.P.); angela.kecskes@aok.pte.hu (A.K.); eva.szoke@aok.pte.hu (É.S.); 2 Department of Chemistry, Durham University, Durham DH1 3LE, UK; david.chisholm@lightox.co.uk (D.R.C.); andy.whiting@durham.ac.uk (A.W.); 3 Department of Pharmacology, Faculty of Pharmacy, University of Pécs, H-7624 Pécs, Hungary

**Keywords:** retinoids, all-trans-retinoic acid, genotoxicity, DNA damage, ATP assay, comet assay

## Abstract

All-trans-retinoic acid (ATRA), the active metabolite of vitamin A, plays a pivotal role in cell differentiation, proliferation and embryonic development. It is an effective therapy for dermatological disorders and malignancies. ATRA is prone to isomerization and oxidation, which can affect its activity and selectivity. Novel diphenylacetylene-based ATRA analogues with increased stability can help to overcome these problems and may offer significant potential as therapeutics for a variety of cancers and neurodegenerative diseases, including amyotrophic lateral sclerosis. Here, we investigated the effects of these retinoids on cell viability and genotoxicity in the widely used model system of the rapidly proliferating Chinese hamster ovary cell line. DC360 is a fluorescent ATRA analogue and DC324 is a non-active derivative of DC360. EC23, DC525, DC540, DC645, and DC712 are promising analogues with increased bioactivity. The cytotoxic activity of the compounds was evaluated by ATP assay and DNA damage was tested by comet assay. No cytotoxicity was observed in the 10^−6^–10^−5^ M concentration range. All compounds induced DNA migration similar to ATRA, but DC324, DC360 and EC23 did so to a greater extent, particularly at higher concentrations. We believe that retinoid receptor-independent genotoxicity is a general characteristic of these compounds; however, further studies are needed to identify the molecular mechanisms and understand their complex biological functions.

## 1. Introduction

Retinoids are vitamin A derivatives that play an important role in cell differentiation, proliferation, and apoptosis [1]. They are approved as chemopreventive and chemotherapeutic agents [2]. However, their use has been limited due to a lack of understanding of their complex signaling pathways and potential systemic toxicity [3,4].

All-trans-retinoic acid (ATRA) is the major vitamin A metabolite [5] and has essential roles in different biological processes during both embryogenesis and adult life [6]. Therefore, it is indicated for the treatment of various skin conditions and cancers, either alone or in combination with other cytostatic drugs. ATRA is prone to isomerization and oxidation, which can lead to significant changes to the compound’s activity and selectivity [6,7]. These changes could be of special interest when studying its toxic effects on non-tumor cells. Synthetic analogues with increased chemical stability could help to overcome these problems and may offer significant potential as pharmaceutical agents with lower toxicity.

The broad side effect profile of retinoids, including mutagenesis, has long held back their clinical use, particularly their systemic administration. The genotoxicity of retinoids is controversial and seems to be dependent on cell types, treatment concentrations and duration, as well as experimental conditions. ATRA did not induce structural aberrations within the chromosomes of human embryonic palatal mesenchymal cells [8]. In contrast, certain retinoids, including 13-cis-retinoic acid, caused sister chromatid exchanges in human diploid fibroblasts [9,10]. In addition, retinol induced chromosomal aberrations in human lymphocytes [11], as well as enhanced DNA single strand breaks and fragmentation, and the formation of 8-oxo-7,8-dihydro-2′-deoxyguanosine, the major DNA oxidation product associated with oxidative stress, in cultured Sertoli cells [12]. Results of a recent clinical trial showed that supplementation with β-carotene (pro-vitamin A) increased the incidence of lung cancer in smokers [13], whereas it had earlier been demonstrated that people with higher serum β-carotene levels had a lower risk of cancer [14]. In addition, ATRA and its steroid analog EA-4 induced micro-nucleation by chromosome breakage in the C2C12 mouse myoblast cell line and HL-60 human acute myeloid leukemia cells [15].

**Figure 1:**
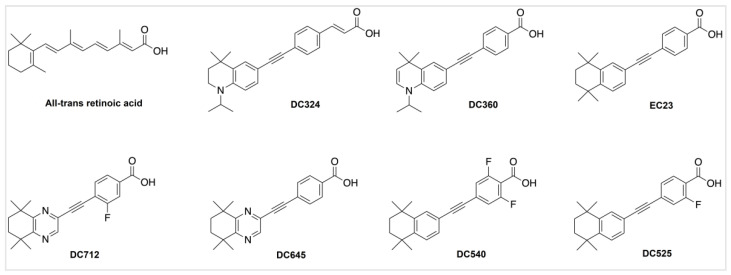
Chemical structures of ATRA and its novel synthetic analogues.

We synthesized novel diphenylacetylene-based ATRA derivatives with a wide range of structural variations (Figure 1) in order to increase their stability and reduce vulnerability to oxidation and isomerization, which might affect their activity and safety. DC360 exhibits fluorescence when activated by visible light (380-420 nm); DC324 is a DC360 derivative with an extended structure that prevents binding to retinoid-related nuclear receptors and transport proteins; hence, it is a non-active fluorescent retinoid [3]. EC23, DC525, DC540, DC645 and DC712 are synthetic ATRA derivatives with increased receptor binding and bioactivity [3,16,17]. These synthetic ATRA analogues were proven to cause differentiation in, for example, neuroblastoma cell lines, similar or more strongly than ATRA [18,19]. They have significant potential as therapeutics for a variety of cancers and neurodegenerative diseases including amyotrophic lateral sclerosis (ALS) [20].

Since there are no data about the potential cytotoxicity and genotoxicity of these novel compounds, here we investigated their effects on cell viability and DNA stability. The aim of this study was to examine the effect of these compounds on normal cells as they have the potential for systemic treatment of various, non-cancerous diseases. Chinese hamster ovary (CHO) cells were chosen, as they represent a widely used model system for the comet assay [21,22,23], which are easy to handle and highly tolerant to variations in pH, oxygen levels and temperature.

## 2. Results

### 2.1. ATRA and Its Synthetic Derivatives Do Not Exert Cytotoxic Effects on CHO Cells

**Figure 2:**
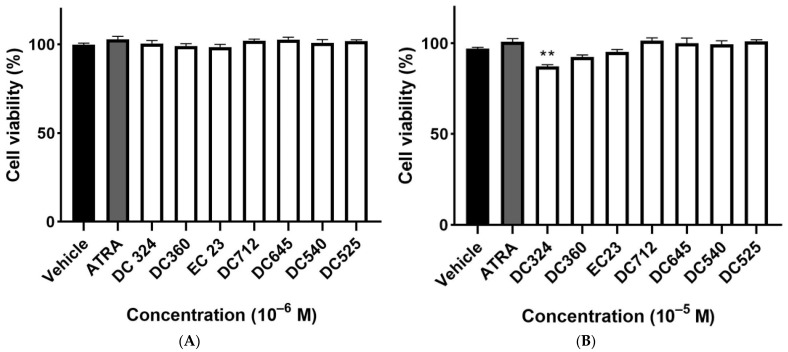
Viability of CHO cells. Cells were exposed to ATRA or its synthetic analogues in 10^−6^ M (**A**) or 10^−5^ M (**B**) for 24 h and cell viability was detected by the ATP viability assay as a percentage of the vehicle-treated control. Each column represents the mean ± SEM of n=12 experiments performed in two independent series (** *p* < 0.01 vs. vehicle).

Neither ATRA nor any of the synthetic retinoic acid derivatives induced cytotoxicity at a concentration of 10^−6^ M (Figure 2A). DC324 exerted a small, but statistically significant decrease in cell viability compared to the vehicle control in 10^−5^ M, but it was still greater than the 70% relative survival that is required for the comet assay (Figure 2B).

### 2.2. Synthetic Derivatives Induce Similar or Greater DNA Damage as Compared to ATRA

**Figure 3:**
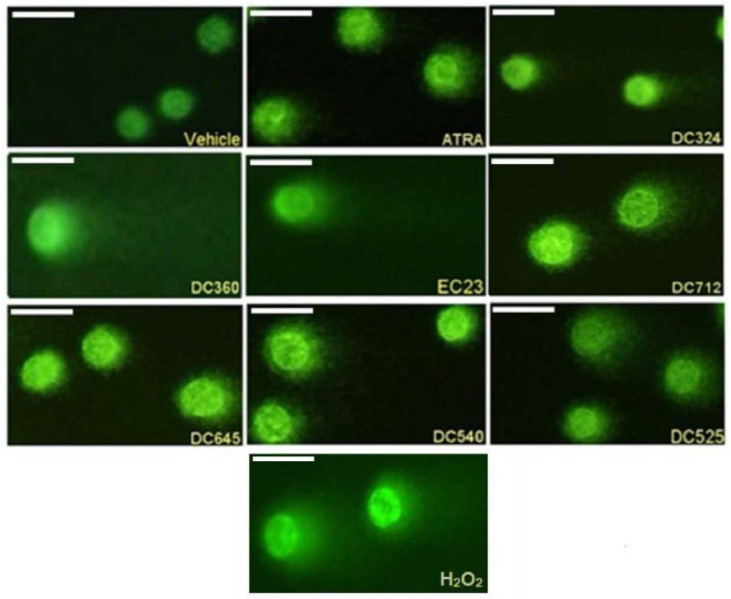
Representative comet images. CHO cells treated for 24 h with vehicle, 10^−5^ M ATRA, DC324, DC360, EC23, DC712, DC645, DC540, DC525, and H_2_O_2_ for 15 min. Magnification 200×.

Representative fluorescence images of CHO cell comets treated with ATRA, DC324, DC360, EC23, DC712, DC645, DC540 and DC525 compared to the vehicle control and H_2_O_2_ in 10^−5^ M concentration are shown in Figure 3.

**Figure 4:**
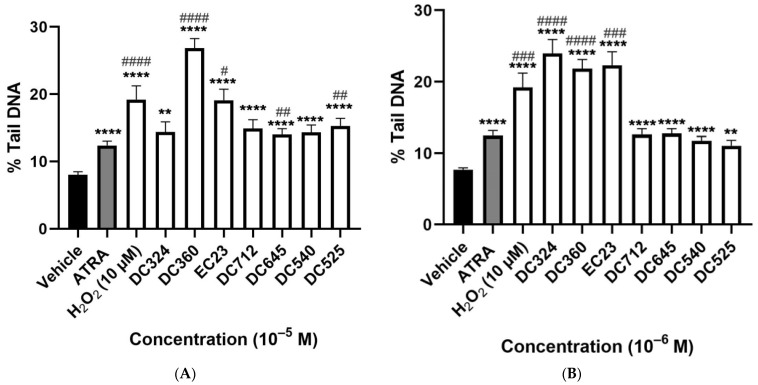

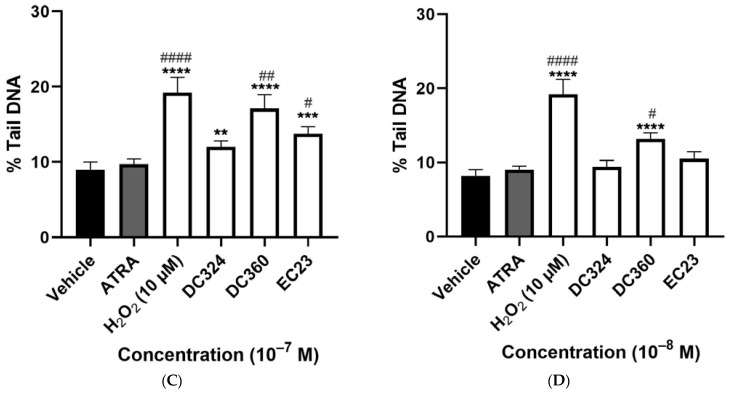
Degree of DNA-damage following retinoid treatments. Columns represent the %tail DNA of CHO cells treated for 24 h with vehicle, 10^−5^ M H_2_O_2_ as the positive control, and the retinoid compounds (10^−5^ M (**A**), 10^−6^ M (**B**), 10^−7^ M (**C**), 10^−8^ M (**D**)). Each column (Except H_2_O_2_) demonstrates the mean ± SEM of n= 100 cells derived from two independent series ( ** *p* < 0.01, *** *p* < 0.001, **** *p* < 0.0001 vs. vehicle), (# *p* < 0.05, ## *p* < 0.01, ### *p* < 0.001, #### *p* < 0.0001 vs. ATRA).

H_2_O_2_ (10^−5^ M) as a positive control significantly increased, by almost double he %tail DNA compared to the vehicle control. ATRA also significantly increased %tail DNA at 10^−5^ and 10^−6^ M but not at lower concentrations; the damaging effect was much less than H_2_O_2_. All tested concentrations of DC360 and the 10^−6^ M concentration of DC324 significantly increased %tail DNA compared to both vehicle control and ATRA at a similar extent to H_2_O_2_ (Figure 4A–D). We observed significant elevation of %tail DNA upon EC23 treatment at a concentration of 10^−7^–10^−5^ M compared to both control and ATRA (Figure 4A–C), but the 10^−8^ M concentration did not induce any alterations (Figure 4D). The highest concentration (10^−5^ M) of DC645 and DC525 significantly elevated %tail DNA compared to both vehicle control and ATRA, while DC712 and DC540 induced a significant increase only compared to vehicle (Figure 4A). At 10^−6^ M, these four compounds showed a significant increase compared to the vehicle control (Figure 4B).

## 3. Discussion

We present here the first functional results for the effects of seven novel diphenylacetylene-based synthetic retinoids on cell viability and DNA stability. It is demonstrated here that these synthetic ATRA analogues do not induce cytotoxic effects, but cause genotoxicity detected by DNA strand breaks in CHO cells. DC360 showed the greatest increase of %tail DNA even at the lowest tested concentration, and the DNA damaging effects of the other derivatives were mostly similar to that of ATRA.

Different in vivo and in vitro studies have shown that retinoids are effective in prevention and treatment of cancer [24,25], but their toxicity has limited their clinical use [26,27]. A strong relationship between DNA damage and cancer has been investigated, where the induction of chromosome breakage is regarded to be genotoxic and potentially carcinogenic [28]. DNA is very sensitive to a wide range of endogenous and exogenous modifications damaging factors leading to mutations [29]. Genomic DNA damage, such as base modifications, strand cross links and breaks occurs spontaneously in response to chemical or physical mutagens [30,31] and influences transcription, replication and chromosome segregation [32]. When there is DNA damage, specific proteins are activated and the cell cycle is arrested until the damage is repaired [33]. If the damage is irreparable, the cells undergo permanent cell-cycle arrest, senescence or cell death (apoptosis/necrosis) [34]. However, when DNA damage is successfully repaired, apoptosis signaling proteins are not activated and the viability of cells is preserved [35]. Therefore, we believe that our compounds cause repairable DNA damage.

The non-cytotoxic concentration was chosen to test the direct effect of the compounds on DNA damage. ATRA and the synthetic analogues did not, at 10^−6^–10^−5^ M concentrations, significantly decrease cell viability, except for DC324, but the inhibition was only 13 ± 1%. This is less than the generally accepted guidelines for the comet assay, which recommend testing above 70% viability [36,37,38]. Therefore, all compounds could be further investigated in the genotoxicity assay.

Our findings regarding the DNA damaging effects of ATRA on CHO cells support previous results in other cell types. For example, ATRA triggered apoptosis in two human hepatoma cell lines, HepG2 and Hep3B, as assessed by flow cytometry [39] and in HL-60 human acute myeloid leukemia cells by typical DNA fragmentation [40]. Moreover, ATRA and its steroid analogue EA-4 caused DNA fragmentation in both C2C12 mouse myocyte cells and HL-60 [41]. ATRA also increased the level of intracellular reactive oxygen species and DNA damage in ARPE-19 cells after exposure to tert-butyl hydroperoxide [42]. All synthetic ATRA analogues induced similar genotoxic effects to ATRA, but DC324, DC360 and EC23 are even more toxic, particularly at higher concentrations. Since the inactive retinoid, DC324, which lacks the ability of retinoid receptor binding, also induced remarkable DNA damage, the observed effects are likely to be independent of the receptor-mediated mechanism. These compounds have the most hydrophobic (high log P) structures and, therefore, they may exhibit stronger off-target interactions with other proteins and DNA species. The enamine function of the dihydroquinoline hydrophobic region of DC360 could also be reactive towards cellular components under certain circumstances; this structural motif may, therefore, be the cause of the stronger genotoxic effect of this compound compared to the others. Further studies are required to elucidate the molecular basis of these genotoxic effects.

Retinol and β-carotene have been proven to have pro-oxidant effects, which might lead to oxidative damage and carcinogenesis [43,44,45]. They can generate free radicals when supplemented at doses higher than the normal dietary intake, which might lead to an increased incidence of different types of cancer, such as esophagus, oral cavity, pharynx, larynx, stomach, colon and rectum [46]. Stimulation of antioxidant enzyme activities and oxidative damage in primary cultured Sertoli cells was observed upon retinol treatment, which may be due to increased iron uptake and storage and the generation of highly reactive hydroxyl radicals by the Fenton reaction [12,47]. This can cause DNA breaks by directly attacking the deoxyribose [48]. Retinol also changes the organization and function of chromatin, thus altering the on- and off-switching of transcriptionally-active regions of DNA in rat Sertoli cells [49], as well as increasing DNA fragmentation in Chinese hamster lung fibroblasts and inducing DNA breaks, cell cycle progression and increasing the numbers of proliferative foci in terminally differentiated rat Sertoli cells [50]. All of these mechanisms might be involved in the observed genotoxic actions of the novel compounds in CHO cells.

The ATP viability assay is a homogenous, widely accepted method as a valid marker of viable cells. It can detect low numbers of cells (10 cells per well) very rapidly, within 10 min to several hours after adding the reagent [51]. Using this assay, we determined the cytotoxicity of our compounds. When the aim is to clarify the mechanism of cytotoxicity, various apoptosis assays can be performed, such as annexin v and caspase assays [52]. In parallel, the comet assay is an efficient and standard method to quantify the extent of DNA damage at the cellular level and is commonly used for genotoxicity and biomonitoring [53]. However, it has some limitations; for example, it only represents the ratio of the fragmented DNA. Therefore, in order to more precisely understand the molecular mechanisms, other assays such as γH2A.X staining and immunoblotting are needed [54]. Here, we screened the effects of our novel compounds on cell viability and DNA fragmentation without the scope of identifying the mechanisms.

In summary, the novel synthetic diphenylacetylene-based ATRA derivatives are not cytotoxic, but they do induce DNA migration due to DNA strand breaks potentially leading to genotoxicity and genome instability. The fluorescent compound, DC360, shows the most pronounced DNA damaging action. Further studies are needed to identify the molecular mechanisms and understand the complex biological activities of these compounds. Based on the present results, we can clearly state that the retinoid receptor-independent genotoxicity is their general characteristic, which should be considered in later development and applications.

## 4. Materials and Methods

### 4.1. Test Compounds

ATRA was purchased from Merck KGaA (Darmstadt, Germany). EC23 and DC324, DC360, DC525, DC540, DC645 and DC712 were prepared according to the published procedures (Figure 4) [3,55,56].

### 4.2. Cell Culture

The Chinese hamster ovary (CHO-K1) cell line (ATCC, Virginia, USA) was chosen as fast-dividing cells (subculturing rate is 1:4–1:8 according to ATCC product sheet). Cells were maintained in Dulbecco’s Modified Eagle’s Medium (DMEM, Thermo Fisher Scientific, Waltham, MA, USA) supplemented with 4 mmol L-glutamine, 10% fetal bovine serum (FBS), 1x penicillin/streptomycin (Thermo Fisher Scientific). Cells were kept at 37 °C in a 5% CO_2_ incubator.

### 4.3. Cell Viability ATP Assay

500 µM stock solutions of compounds in absolute ethanol (vehicle) were initially prepared. CellTiter-Glo^®^ Luminescent Cell Viability Assay (Promega, Mannheim, Germany) was used to determine the number of viable CHO cells in culture. CHO cells were seeded in a 96-well plate at a density of 5000 cells/well in Dulbecco’s Modified Eagle’s Media supplemented with 10% FBS, 4 mmol *L*-glutamine and kept in incubator at 37 °C and 5% CO_2_. After 24 h, cells were treated with 10^−6^ M and 10^−5^ M retinoic acid derivatives (prepared in 100 µL of media) and incubated for 24 h. After incubation, 100 µL CellTiter-Glo reagent was added to each well. The plate was placed for 2 min onto an orbital shaker. After 10 min incubation at room temperature, cell viability was measured with a luminescence microplate reader (EnSpire AlphaLISA, PerkinElmer, Inc, Waltham, MA, USA) at an integration time of 0.25–1 s per well. Vehicle treated wells served as negative control. The viability of treated cells was determined by comparing ATP levels of treated wells to vehicle-treated controls. The experiment was repeated twice with 6 wells/compound each time.

### 4.4. DNA Damage Comet Assay (Single Cell Gel Electrophoresis)

The comet assay is a fundamental method to detect DNA damage. Cells embedded on a slide are lysed to form a nucleoid consisting of a DNA dense core surrounded by a lighter halo [57]. The presence of strand breaks relaxes the DNA supercoiling forming a loop, which is released into the halo during alkaline electrophoresis [58] resulting in comet-like structures [59]. The number of relaxed loops in the tail indicates the number of DNA breaks [38].

Initially, the CHO cells were cultured in 6-well plate Frosted microscopic slides were coated with 0.8% normal melting point agarose (SeaKem^®^, Lonza) and stored for future use. Cells were treated for 24 h with ATRA, DC324, DC360, EC23, DC712, DC645, DC540, DC525 or hydrogen peroxide (H_2_O_2_) for 15 min as positive control; vehicle-treated wells were used as negative controls. The tested concentrations of compounds were chosen as non-cytotoxic. In brief, cells were trypsinized, centrifuged, resuspended and mixed well with 0.7% low melting point agarose at 40 °C. A 200 μL sample was transferred onto the pre-coated slide (3 slides/treatment) and covered with a coverslip. Slides were placed at 4 °C for 5 min to allow agarose solidification. Then, coverslips were removed, and slides were incubated in a lysis solution (pH 10) at 4 °C overnight. Electrophoresis was performed at 25 V, 300 mA for 30 min in a cold electrophoresis buffer (pH 13). Slides were then washed with neutralizing buffer (pH 7.5), distilled water, and absolute ethanol (5 min in each solution). Finally, slides were allowed to dry, and then stained with Eco-safe (PacificImage Electronics) stain in the dark at RT. After the removal of excess stain, the slides were covered with 2–3 drops of fluoromount solution (Fluoromount-G™, Invitrogen) and a coverslip. Image acquisition was performed using Olympus BX50 fluorescence microscope with 200× magnification and evaluated with an image analysis software program (OpenComet plugin in ImageJ software). Two independent experiments were conducted for each treatment in which three slides were used to score different cells. At least 50 cells/ experiment (except for H_2_O_2_) were selected randomly for the analysis of comet by quantifying the DNA damage as total percentage of DNA in the tail (%tail DNA).

### 4.5. Statistical Analysis

Statistical analysis was performed by Graphpad Prism (Version 8.0.1). The normality was tested by Shapiro–Wilk test, and, since data were not normally distributed, they were analyzed using the Kruskal–Wallis followed by Dunn’s multiple comparisons test. The results are presented as mean ± SEM at α = 0.05 level of significance.

## Data Availability

Not applicable.

## Sample Availability

Samples of the compounds are available from the authors upon suitable request.
